# Efficacy and Safety of Ginkgo Leaf Extract and Dipyridamole Injection for Ischemic Stroke: A Systematic Review and Meta Analysis

**DOI:** 10.3389/fphar.2019.01403

**Published:** 2019-12-04

**Authors:** Ping Xue, Zhuoya Ma, Shuguang Liu

**Affiliations:** ^1^Department of Neurology, Liaocheng People’s Hospital, Liaocheng, China; ^2^Department of Intensive Care Unit, Yanggu People’s Hospital, Yanggu, China

**Keywords:** ginkgo leaf extract and dipyridamole injection, traditional Chinese medicine, conventional treatments, ischemic stroke, meta-analysis

## Abstract

**Objective:** Ginkgo leaf extract and dipyridamole injection (GDI), a kind of Chinese medicine preparation, has been considered as a promising supplementary treatment for ischemic stroke. The aim of this study was to systematically evaluate the clinical efficacy and safety of GDI mediated therapy for ischemic stroke.

**Methods:** PubMed, Cochrane Library, Medline, Embase, Web of Science, Wanfang database, Chinese Scientific Journal Database (VIP), China National Knowledge Infrastructure (CNKI) and Chinese Biological Medicine Database (CBM), were searched systematically for clinical trials of conventional treatments combined with GDI for ischemic stroke. The reported outcomes including overall response, hemorrheology and blood lipid indexes, and adverse events were systematically investigated.

**Results:** Data from thirty-nine trials including 3,182 ischemic stroke patients were involved. The results indicated that, compared with conventional treatments alone, the combination of conventional treatments with GDI obviously improved the overall response (odds ratio [OR] = 4.14, 95% confidence interval [CI] = 3.26–5.25, *P* < 0.00001), neurological status (National Institutes of Health Stroke Scale, OR = −3.13, 95% CI = −3.98 to −2.28, *P* < 0.00001) and activity of daily living (Barthel Index score, OR = 14.10, 95% CI = 9.51–18.68, *P* < 0.00001) of patients. Moreover, the hemorheology and blood lipids indexes of ischemic stroke patients were also significantly ameliorated after the combined therapy (*P* < 0.01). The frequency of adverse events did not differ significantly between the two groups (*P* > 0.05).

**Conclusion:** Evidence from the meta-analysis suggested that the combination of conventional treatments and GDI is safe and more effective in treating ischemic stroke than conventional treatments alone. Therefore, GDI mediated therapy could be recommended as an adjuvant treatment for ischemic stroke.

## Introduction

Ischemia stroke is one of the common cerebrovascular diseases, and is a major cause of death and disability (Chen et al., 2019). It is characterized by the partial or complete loss of blood supply in part of the brain tissues, which account for about 80% of all stroke events (Chen et al., 2019). Ischemia results in reduced neuron number and interrupted neural axon network, and eventually resulting in the permanent loss of nerve tissue or disabled brain function (Xue et al., 2018). Over fifty million peoples are suffering ischemic stroke in the world, and nearly 50% of stroke survivors are left with disabling sequelae (Xue et al., 2018). Currently, the conventional therapy, including thrombolysis, controlling cerebral edema, neuroprotective agents, restoring blood supply to ischemic area, reducing blood viscosity, preventing and treating complications, controlling hypertension, etc. is the main clinical therapy for ischemia stroke (Ma et al., 2017; Chen et al., 2019). However, it functions mainly at the early stage of ischemia with a short time window, and therefore its clinical application is severely limited (Xue et al., 2018). Thus, the more effective agents for ischemia stroke patients are desirable.

Ginkgo extract is a Chinese traditional herb which made from the dried leaves of the *Ginkgo biloba* L (Ginkgoaceae) (Zeng et al., 2005), and has been widely applied as an effective complementary drug for brain disorder treatment in numerous hospitals of China. Ginkgo extract can protect the neurons against reactive oxygen species, regulates vasomotor, improves hemorheology and can also reduces infarction size by improving neurological function (Kampkotter et al., 2007; Nash and Shah, 2015; Tulsulkar et al., 2016; Liu et al., 2019). Zhang et al. (2018) demonstrated that ginkgo extract can inhibit astrocytic lipocalin-2 expression and alleviates neuro-inflammatory injury *via* the JAK2/STAT3 pathway after ischemic stroke. Ginkgo leaf extract and dipyridamole injection (GDI) is a compound preparation, which mainly consists of ginkgo flavone glycosides (24–25%), terpene lactones [ginkgolides (3.1%) and bilobalide (2.9%)] and dipyridamole (10%) (Zeng et al., 2005; Tan et al., 2018). Currently, several clinical trials indicated that conventional treatments combined with GDI exhibits more prominent therapeutic effects for ischemic stroke than conventional treatments alone (Wang et al., 2015; Li et al., 2017). However, the scientific evidence has not been systematically reviewed. Therefore, in this study, we conducted a meta-analysis to investigate the clinical efficacy and safety of GDI for ischemic stroke, in order to provide the best available evidence for clinical practice and further research planning on ischemia stroke treatment.

## Materials and Methods

### Search Strategy and Selection Criteria

This systematic review and meta-analysis was performed following the PRISMA guidelines and Cochrane Handbook. Literatures were searched across nine electronic databases, including PubMed, Cochrane Library, Medline, Embase, Web of Science, Wanfang database, Chinese Scientific Journal Database (VIP), China National Knowledge Infrastructure (CNKI) and Chinese Biological Medicine Database (CBM), before December 2018, with key terms “ginkgo biloba” or “ginkgo leaf extract” or “ginkgo dipyidamolum” or “ginkgo leaf extract and dipyridamole injection” or “yinxingdamo injection” and “ischemic stroke” or “cerebral infarction” or “brain infarction” or “cerebral ischemia” or “brain ischemia,” without language restriction (**Supplementary Table 1**). No language limits were applied.

Inclusion criteria: (1) Randomized controlled trials (RCTs) concerning ischemic stroke patients were included; (2) Research subjects (ischemic stroke patients) must meet WHO diagnostic criteria of ischemic stroke and exclude cerebral hemorrhage by brain computerized tomography (CT) or magnetic resonance imaging (MRI). (3) Articles involving more than 30 ischemic stroke patients; (4) There were no other medicines in combination with the conventional treatments in the experimental group, except for GDI, compared with the conventional treatments as a control; (5) Literatures comparing the clinical outcomes of conventional treatments plus GDI adjuvant therapy (experimental group) with conventional treatments alone (control group); (6) One or more outcome measures, including the overall response rate, neurological deficit score, serum level of CRP, hemorheology and blood lipid indexes, adverse events must be included in each study.

Exclusion criteria: (1) Studies not focus on GDI were excluded; (2) Inappropriate criteria in experimental or control group were excluded; (3) articles without sufficient available data were excluded; (4) Non-contrast articles, non-clinical studies, literature reviews, meta-analysis, meeting abstracts, case reports, repeated studies and experimental model researches were excluded.

### Data Extraction and Quality Assessment

Data were extracted by two reviewers (PX and ZM) independently according to the same inclusion and exclusion criteria; disagreements were adjudicated by the third investigator (SL). The extracted characteristics comprised the following items: (a) first author’s names; (b) years of publication; (c) number of cases; (d) patient ages; (e) intervening measure; (f) dosage of GDI; (g) duration of treatment and (h) study parameter types. The quality of included trials was evaluated according to Cochrane Handbook (Zeng et al., 2015).

### Outcome Definition

Clinical responses include treatment efficacy and adverse events. Treatment efficacy was evaluated in terms of the overall response rate (ORR), National Institutes of Health Stroke Scale (NIHSS), Barthel Index (BI) score, hemorrheology and blood lipid indexes. The hemorrheology indexes covered the following indicators: whole blood viscosity (WBV), plasma viscosity (PV), whole blood high-shear viscosity (WBHSV), whole blood low-shear viscosity (WBLSV), and content of fibrinogen (FIB). The blood lipid indicators [Plasma total cholesterol (TC), triglycerides (TG), high density lipoprotein-cholesterol (HDL-C); low density lipoprotein-cholesterol (LDL-C)] and plasma C reactive protein (CRP) of ischemic stroke patients were determined and compared between the GDI and non-GDI groups. Adverse events including fever, fullness in head, allergy, hemorrhage, palpitation, nausea and vomiting were taken into assessment.

### Statistical Analysis

Statistical analysis was performed using the Review Manager 5.3 (Cochrane Collaboration, Oxford, UK) and Stata 13.0 (Stata Corp., College Station, TX, USA). All data were expressed as odds ratio (OR) and 95% confidence intervals (CI), and *P* < 0.05 indicates difference with statistical significance. Heterogeneity among studies was estimated using the Cochran’s Q statistic and *I* ^2^ tests, *I* ^2^ > 50% or *P* < 0.1 indicated a high statistical heterogeneity (Jackson et al., 2012). A fixed-effects model was used to pool the estimates when heterogeneity was absent (*I* ^2^ < 50%). Otherwise, a random effects model was selected.

Publication bias was numerically examined by Begg’s and Egger’s tests and presented by funnel plots. If publication bias existed, we used the trim-and-fill method to adjust the pooled estimates of the potential unpublished studies in the meta-analysis, which were compared with the original pooled OR (Liang et al., 2017; Lin et al., 2018). Sensitivity analysis was conducted to investigate the influence of different GDI dosages, sample sizes and research types on clinical efficacy.

## Results

### Search Results

**Figure 1:**
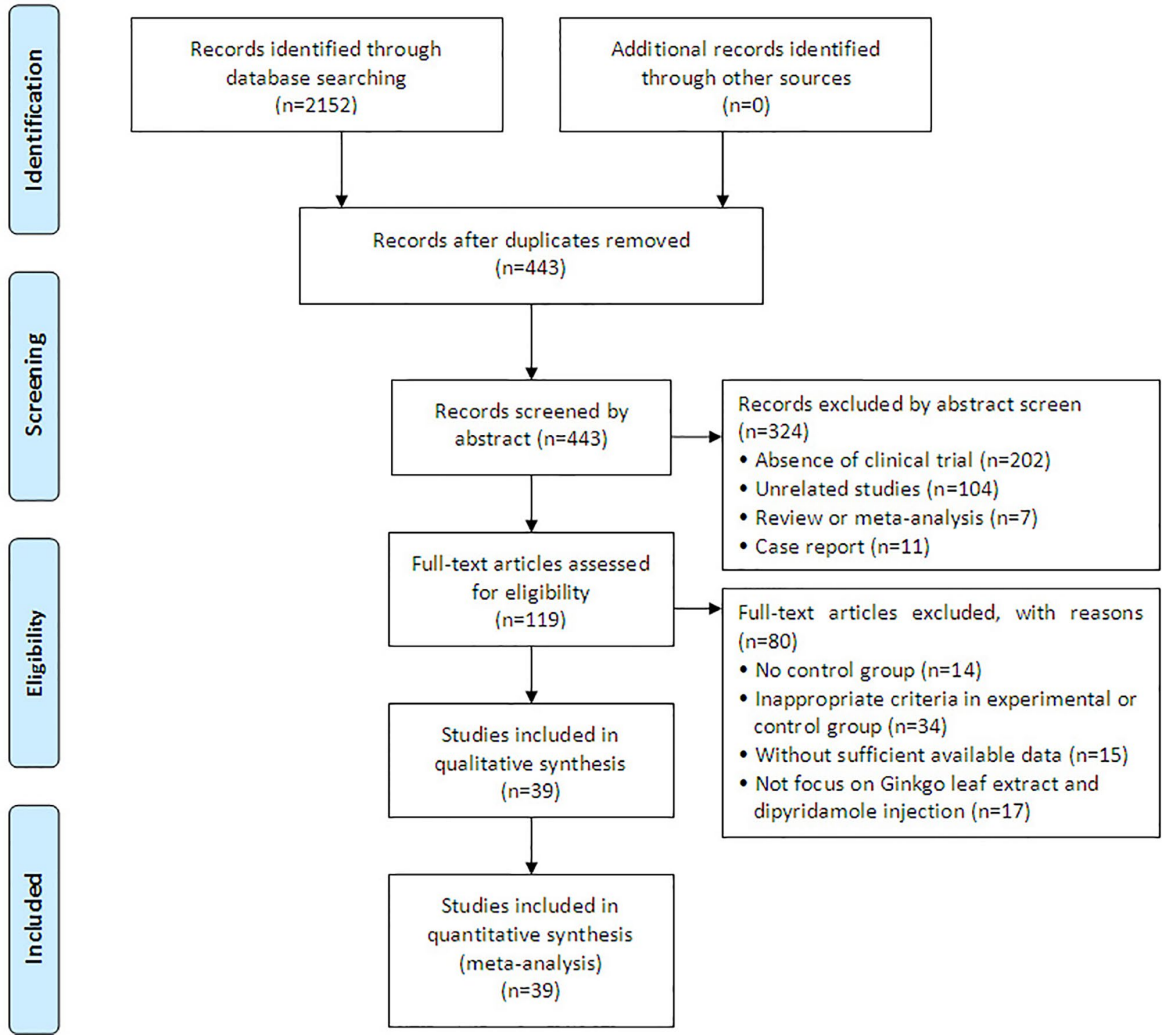
Study selection process for the meta-analysis.

A total of 2,152 articles were identified with initial retrieve. 1,709 papers were excluded due to duplication. After title and abstract review, 324 articles were further excluded because they were not clinical trials (n = 202) or were unrelated studies (n = 104) or were reviews and meta-analysis (n = 7) or were case report (n = 11), leaving 119 studies as potentially relevant. After detailed assessment of full texts, articles without control group (n = 14), publications with inappropriate criteria of experimental or control group (n = 34), trials with insufficient data (n = 15) and studies not focus on GDI (n = 17) were excluded. Finally, 39 trials (Tang et al., 2009; Wang et al., 2009; Li, 2010; Long et al., 2010; Huang and Yuan, 2010; Tian et al., 2010; Zhou et al., 2010; Chen et al., 2011; Yang, 2012; Wang, 2012; Lan, 2013; Ding, 2013; Lin and Lin, 2013; Tang, 2013; Fang, 2014; Huang et al., 2014; Jiang, 2014; Yang, 2014; Wang, 2014a; Wang, 2014b; Chu, 2015; Liu, 2015; Sun, 2015; Zeng, 2015; Chen, 2016; Cui, 2016; Fu et al., 2016; Zhou et al., 2016; Li, 2016; Li, 2017; Dai, 2017; Guo, 2017; Wei, 2017; Ai, 2017; Zhang, 2017; Wang, 2018; Yi et al., 2018; Zhang, 2018; Zheng et al., 2018) involving 3,182 ischemic stroke patients were included in this analysis (**Figure 1**).

### Patient Characteristics

After selection, all included trials were performed in different medical centers of China. In total, 1,596 ischemic stroke patients were treated by conventional treatments in combination with GDI adjuvant therapy, while 1,589 patients were treated by conventional treatments alone. Detailed information of the involved studies and ischemic stroke patients is shown in **Table 1**. All included trials except one (Yang, 2014) clearly introduce the dosage of GDI. Twenty-two studies specifically describe the manufacturer of GDI and the remaining seventeen studies lacked clear description of production information (**Supplementary Table 2**). The compositions and concentrations of GDI in all included trials are the same (every 10ml GDI contained 9.0–11.0 mg total flavonoids and 3.6–4.4 mg dipyridamole). The Quality Standards of GDI in this study have been approved by Chinese State Food and Drug Administration (SFDA), and granted the Manufacturing Approve Number issued by Chinese SFDA. All pharmaceutical companies involved followed the quality processing procedure outlined in pharmacopeia.

**Table 1:** Clinical information from the eligible trials in the meta-analysis.

Included studies	Patients Con/Exp	Age (year)		Interview Measure(Exp/Con)	Conventional treatments	Dosage of GDI	Duration	Parameter types
		Con	Exp					
Ai DJ (2017)	34/34	–	–	CT+GDI vs CT	Ganglioside	20-30 ml/Day	–	ORR
Chen L (2011)	30/32	61 ± 5.87 (mean)	61 ± 5.32 (mean)	CT+GDI vs CT	–	20 ml/Day	14 days	ORR, BVI
Chen TH (2016)	31/31	58 ± 1.36 (mean)	59 ± 2.13 (mean)	CT+GDI vs CT	–	20 ml/Day	30 days	ORR, NIHSS, CRP
Chu WM (2015)	30/30	66.38 ± 7.57 (mean)	67.17 ± 5.34 (mean)	CT+GDI vs CT	–	30 ml/Day	14 days	NIHSS, BLI
Cui XF (2016)	35/35	–	–	CT+GDI vs CT	Ozagrel	40 ml/Day	28 days	ORR
Dai CM (2017)	50/50	–	–	CT+GDI vs CT	Aspirin	20 ml/Day	14 days	ORR
Ding HY (2013)	30/30	75.6 ± 5.3 (mean)	75.3 ± 5.1 (mean)	CT+GDI vs CT	Ganglioside	30 ml/Day	14 days	BVI, AE
Fang XW (2014)	50/50	–	–	CT+GDI vs CT	–	20 ml/Day	28 days	ORR, CRP
Fu DF (2016)	42/42	61.7 (mean)	61.5 (mean)	CT+GDI vs CT	–	20 ml/Day	14 days	ORR, NIHSS, BI
Guo ZX (2017)	33/35	60 (mean)	62 (mean)	CT+GDI vs CT	Ozagrel	20 ml/Day	14 days	ORR, AE
Huang SP (2010)	42/42	66.9 (mean)	67.8 (mean)	CT+GDI vs CT	–	40 ml/Day	–	BI
Huang WH (2014)	39/39	58.94 ± 5.28 (mean)	57.63 ± 5.73 (mean)	CT+GDI vs CT	Ozagrel	20 ml/Day	14 days	ORR, AE
Jiang X (2014)	50/50	–	–	CT+GDI vs CT	Aspirin	20 ml/Day	14 days	ORR, NIHSS, BVI
Lan BW (2013)	35/35	52.71 ± 3.35 (mean)	52.32 ± 3.11 (mean)	CT+GDI vs CT	Ozagrel	20 ml/Day	14 days	ORR, AE
Li CX (2010)	43/43	–	–	CT+GDI vs CT	–	20 ml/Day	14 days	CRP
Li GQ (2017)	43/43	–	–	CT+GDI vs CT	Edaravone	25 ml/Day	14 days	ORR, AE
Li NP (2016)	30/30	65.0 ± 7.5 (mean)	66.9 ± 6.8 (mean)	CT+GDI vs CT	–	20 ml/Day	14 days	ORR, NIHSS, CRP, BVI
Lin CD (2013)	32/32	–	–	CT+GDI vs CT	Cinepazide malete	20 ml/Day	28 days	ORR
Liu W (2015)	17/18	60.5 ± 0.5 (mean)	70.8 ± 0.8 (mean)	CT+GDI vs CT	Ozagrel	20 ml/Day	15 days	ORR, AE
Long XY (2010)	75/76	64.2 (median)	65.4 (median)	CT+GDI vs CT	Heparin	20 ml/Day	14 days	ORR, BVI, AE
Sun YF (2015)	38/41	65.7 ± 2.3 (mean)	64.3 ± 2.4 (mean)	CT+GDI vs CT	Edaravone	20 ml/Day	14 days	ORR, NIHSS, AE
Tang HM (2009)	45/45	–	–	CT+GDI vs CT	Aspirin	20 ml/Day	14 days	ORR, BVI
Tang XJ (2013)	39/39	–	–	CT+GDI vs CT	Aspirin	20 ml/Day	14 days	ORR, NIHSS, BVI
Tian XJ (2010)	33/34	66.4 (mean)	67.5 (mean)	CT+GDI vs CT	Fibrinolytic enzyme	20 ml/Day	14 days	ORR, NIHSS, BLI
Wang FF (2014)	30/30	52-65	50-62	CT+GDI vs CT	Ozagrel	20 ml/Day	14 days	ORR, AE
Wang JS (2009)	50/50	59 (median)	61 (median)	CT+GDI vs CT	Nimodipine	20 ml/Day	14 days	ORR, CRP, AE
Wang Q (2014)	60/60	–	–	CT+GDI vs CT	Aspirin	20 ml/Day	14 days	ORR, AE
Wang YH (2012)	59/60	60 ± 9.78 (mean)	59 ± 10.9 (mean)	CT+GDI vs CT	Aspirin	30 ml/Day	14 days	ORR
Wang ZG (2018)	18/18	65.63 ± 8.05 (mean)	65.17 ± 8.54 (mean)	CT+GDI vs CT	Ozagrel	20 ml/Day	14 days	ORR, NIHSS
Wei N (2017)	61/61	65.93 ± 5.52 (mean)	66.26 ± 5.47 (mean)	CT+GDI vs CT	Edaravone	10 ml/Day	14 days	ORR, BVI
Yang J (2012)	29/27	55.4 ± 17.6 (mean)	60.5 ± 16.4 (mean)	CT+GDI vs CT	–	30 ml/Day	15 days	ORR
Yang L (2014)	37/37	–	–	CT+GDI vs CT	Aspirin	–	14 days	ORR, NIHSS, BVI
Yi JT (2018)	45/45	54.12 ± 12.25 (mean)	53.74 ± 10.78 (mean)	CT+GDI vs CT	–	25 ml/Day	14 days	ORR, NIHSS, BVI
Zeng JW (2015)	27/28	58.9 ± 3.9 (mean)	59.5 ± 4.1 (mean)	CT+GDI vs CT	Ozagrel	20 ml/Day	14 days	ORR, AE
Zhang SY (2017)	45/45	60.12 ± 2.36 (mean)	60.13 ± 2.31 (mean)	CT+GDI vs CT	Ozagrel	30 ml/Day	14 days	ORR, NIHSS, BVI, AE
Zhang T (2018)	50/50	43.23 ± 1.34 (mean)	42.96 ± 1.48 (mean)	CT+GDI vs CT	–	30 ml/Day	14 days	ORR, NIHSS, CRP, BI
Zheng XH (2018)	50/50	64.1 ± 3.0 (mean)	68.2 ± 3.4 (mean)	CT+GDI vs CT	GM1	20 ml/Day	14 days	ORR, BLI
Zhou J (2016)	39/39	64.8 ± 11.1 (mean)	64.2 ± 11.2 (mean)	CT+GDI vs CT	Ozagrel	40 ml/Day	14 days	ORR, NIHSS
Zhou M (2010)	60/60	64.3 ± 7.9 (mean)	62.5 ± 6.8 (mean)	CT+GDI vs CT	–	20 ml/Day	14 days	ORR

Con, control group (conventional treatments alone group); Exp, experimental group (conventional treatments and GDI combined group). ORR, overall response rate; CT, conventional treatments; GDI, Ginkgo leaf extract and dipyridamole injection; GM1, monosialotetrahexosylganglioside; NIHSS, National Institutes of Health Stroke Scale; BI, Barthel Index score; BVI, Blood Viscosity Index; BLI, Blood lipid index; CPR, plasma C reactive protein; AE, adverse events.

### Quality Assessment

**Figure 2:**
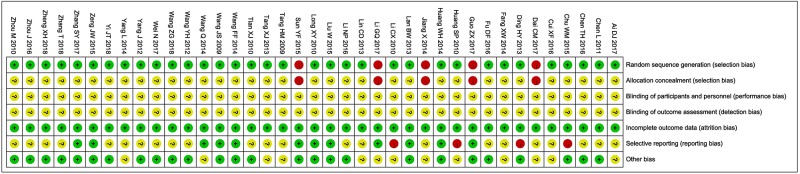
Risk of bias summary: review of authors’ judgments about each risk of bias item for included studies. Each color represents a different level of bias: red for high-risk, green for low-risk, and yellow for unclear-risk of bias.

The assessment of bias risk is shown in **Figure 2**. Thirty-four studies (Wang et al., 2009; Tang et al., 2009; Li, 2010; Huang and Yuan, 2010; Tian et al., 2010; Long et al., 2010; Zhou et al., 2010; Chen et al., 2011; Wang, 2012; Yang, 2012; Ding, 2013; Lin and Lin, 2013; Tang, 2013; Lan, 2013; Yang, 2014; Huang et al., 2014; Fang, 2014; Wang, 2014a; Wang, 2014b; Chu, 2015; Liu, 2015; Zeng, 2015; Cui, 2016; Fu et al., 2016; Li, 2016; Zhou et al., 2016; Chen, 2016; Zhang, 2017; Wei, 2017; Ai, 2017; Yi et al., 2018; Wang, 2018; Zhang, 2018; Zheng et al., 2018) were determined as low risk and the remaining five studies (Jiang, 2014; Sun, 2015; Dai, 2017; Guo, 2017; Li, 2017) were not true RCTs. All included trials did not provide clear description of performance and detection risks. The attrition risks of involved trials were low. Four trials (Huang and Yuan, 2010; Li, 2010; Ding, 2013; Chu, 2015) were considered as high reporting risk owing to lack of primary outcomes (ORR) and twenty-three studies (Tang et al., 2009; Zhou et al., 2010; Tian et al., 2010; Chen et al., 2011; Wang, 2012; Yang, 2012; Tang, 2013; Lin and Lin, 2013; Yang, 2014; Jiang, 2014; Fang, 2014; Fu et al., 2016; Li, 2016; Chen, 2016; Cui, 2016; Zhou et al., 2016; Ai, 2017; Dai, 2017; Wei, 2017; Zhang, 2018; Zheng et al., 2018; Wang, 2018; Yi et al., 2018) were considered as unclear reporting risk due to lack of safety assessment.

### ORR Assessments

**Figure 3:**
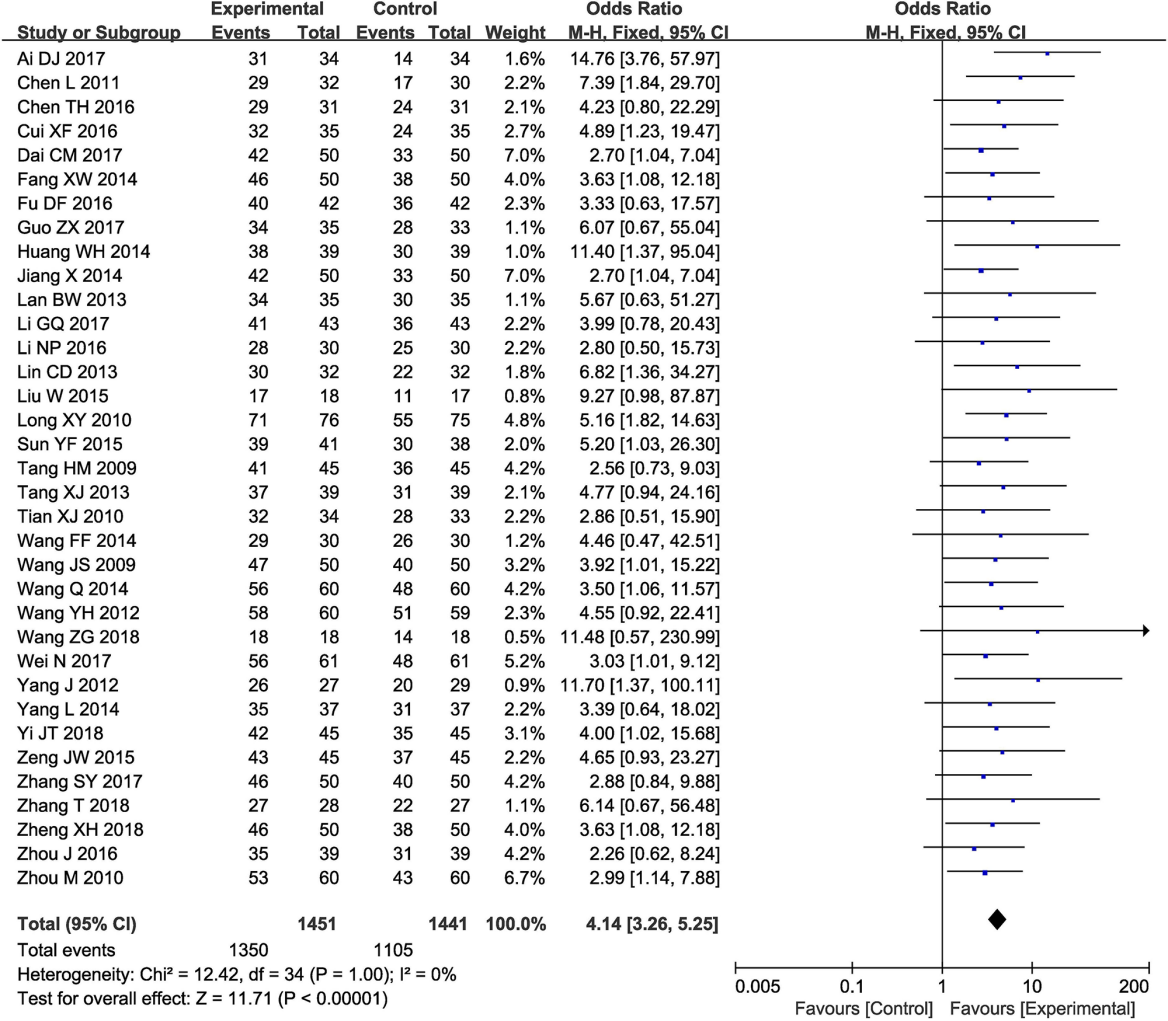
Forest plot of the comparison of overall response rate (ORR) between the experimental and control group. Control group, conventional treatments alone group; Experimental group, conventional treatments and GDI combined group. GDI, Ginkgo leaf extract and dipyridamole injection. The fixed-effects meta-analysis model (Mantel-Haenszel method) was used.

Thirty-five clinical trials (Wang et al., 2009; Tang et al., 2009; Tian et al., 2010; Long et al., 2010; Zhou et al., 2010; Chen et al., 2011; Yang, 2012; Wang, 2012; Lan, 2013; Tang, 2013; Lin and Lin, 2013; Huang et al., 2014; Jiang, 2014; Wang, 2014a; Wang, 2014b; Fang, 2014; Yang, 2014; Liu, 2015; Sun, 2015; Zeng, 2015; Chen, 2016; Cui, 2016; Li, 2016; Zhou et al., 2016; Fu et al., 2016; Dai, 2017; Guo, 2017; Li, 2017; Wei, 2017; Zhang, 2017; Ai, 2017; Wang, 2018; Yi et al., 2018; Zhang, 2018; Zheng et al., 2018) involving 2,792 cases compared the ORR between the two groups (**Figure 3**). Our pooled results showed that patients underwent combined therapy had significantly improved ORR (OR = 4.14, 95% CI = 3.26-5.25, *P* < 0.00001) compared with conventional treatments alone. There was no heterogeneity, and a fixed-effect model was used to carry out the meta-analysis.

### NIHSS

**Figure 4:**
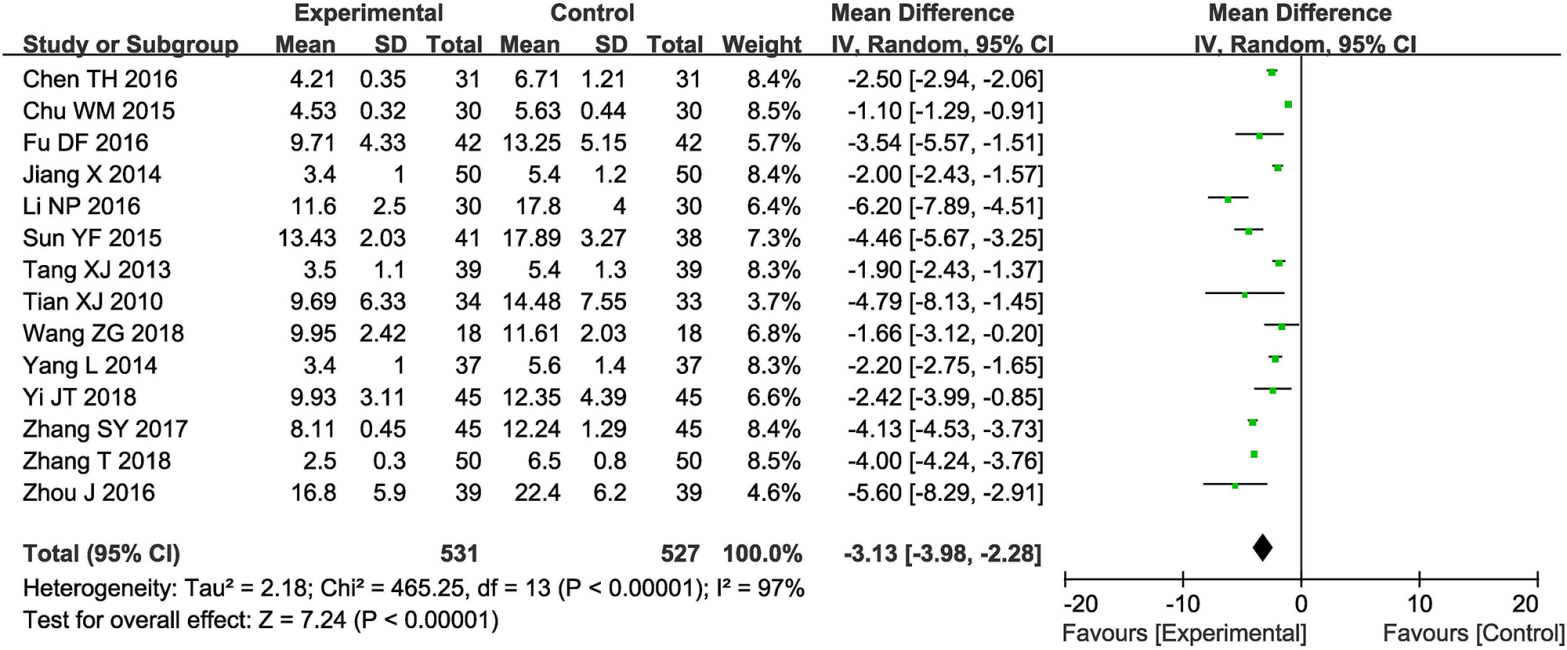
Forest plot of the comparison of National Institutes of Health Stroke Scale (NIHSS) between the experimental and control group. Control group, conventional treatments alone group; Experimental group, conventional treatments and GDI combined group. GDI, Ginkgo leaf extract and dipyridamole injection. The random effects meta-analysis model (Inverse Variance method) was used.

Fourteen trials (Tian et al., 2010; Tang, 2013; Jiang, 2014; Yang, 2014; Chu, 2015; Sun, 2015; Chen, 2016; Fu et al., 2016; Li, 2016; Zhou et al., 2016; Zhang, 2017; Zhang, 2018; Yi et al., 2018; Wang, 2018) with 1,058 participants measured the neurological status according to the NIHSS (**Figure 4**). Results showed that the neurological status of ischemic oke patients received combined therapy was obviously improved compare to those treated by conventional treatments alone (OR = −3.13, 95% CI = −3.98 to −2.28, *P* < 0.00001). There was significant heterogeneity among the studies (I^2^ = 97%, *P* < 0.00001); therefore, a random-effects model was conducted to pool data and so any conclusions need to be made with caution.

Sensitivity Analysis Was Performed to Explore an Individual Study’s Influence on the Pooled Results by Deleting One Single Study Each Time From Pooled Analysis. as **Supplementary Table 3** and **Supplementary Figure 1** Signified, the Results Revealed That No Individual Studies Significantly Affected the NIHSS, Which Indicated Statistically Robust Results.

### BI Score

**Figure 5:**
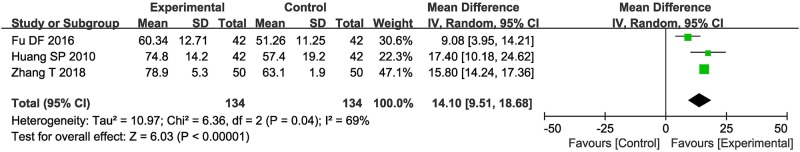
Forest plot of the comparison of Barthel Index (BI) score between the experimental and control group. Control group, conventional treatments alone group; Experimental group, conventional treatments and GDI combined group. GDI, Ginkgo leaf extract and dipyridamole injection. The random effects meta-analysis model (Inverse Variance method) was used.

Three trials (Huang and Yuan, 2010; Fu et al., 2016; Zhang, 2018) involving 368 ischemic stroke patients evaluated the activity of daily living according to the BI Score. As shown in **Figure 5**, the BI Score of ischemic stroke patients in the combined group were significantly higher than that of the control group (OR = 14.10, 95% CI = 9.51–18.68, *P* < 0.00001). A *P*-value = 0.04 and I^2^ = 69% indicated that there was significant heterogeneity among the studies; thus a random effect model was employed.

### CRP Level

There were six studies (Wang et al., 2009; Li, 2010; Fang, 2014; Li, 2016; Chen, 2016; Zhang, 2018) involving 508 patients measured the level of CRP (**Supplementary Figure 2**). The pooled analysis showed that compared with the conventional treatments alone, combined with GDI could significantly reduce the serum CRP levels of ischemic stroke patients (OR = −2.36, 95% CI = −3.25 to −1.48, *P* < 0.00001). There was statistical heterogeneity in CRP (*P* < 0.00001, I^2^ = 92%) according to the heterogeneity test. Therefore, the random effects model was used to pool this meta-analysis.

### Hemorrheology Assessment

The hemorrheology of ischemic stroke patients was measured between GDI and non-GDI groups in eleven controlled studies (Tang et al., 2009; Long, et al., 2010; Chen, et al., 2011; Ding, 2013; Tang, 2013; Jiang, 2014; Yang, 2014; Li, 2016; Wei, 2017; Zhang, 2017; Yi et al., 2018) (**Supplementary Figure 3**). In this analysis, our results showed that the hemorrheology of ischemic stroke patients received combined therapy was significantly improved compare to those treated by conventional treatments alone, indicated by significantly reduced WBV (OR = −1.32, 95% CI = −1.40 to −1.24, *P* < 0.00001), PV (OR = −0.34, 95% CI = −0.51 to −0.17, *P* < 0.0001), WBHV (OR = −1.64, 95% CI = −2.39 to −0.89, *P* < 0.0001), WBLV (OR = −1.56, 95% CI = −2.33 to −0.79, *P* < 0.0001) and FIB (OR = −0.55, 95% CI = −1.43 to −0.34, *P* = 0.002). WBV (*P* = 0.99, I^2^ = 0%) was not heterogeneous among the studies, so fixed-effect model was used to analyzing its OR. Otherwise, random-effect model was used.

### Blood Lipid Assessment

There were three studies (Tian et al., 2010; Chu, 2015; Zheng et al., 2018) reported the amelioration of blood lipid after the treatment of GDI and conventional therapy (**Supplementary Figure 4**). The meta-analysis revealed that the blood lipid level of patients was significantly improved compared with the conventional treatments alone, indicated by obviously decreased levels of TC (OR = −0.65, 95% CI = −0.82 to −0.48, *P* < 0.00001), TG (OR = −0.86, 95% CI = −1.24 to −0.49, *P* < 0.00001) and LDL-C (OR = −0.97, 95% CI = −1.24 to −0.70, *P* < 0.00001), and significantly increased HDL-C levels (OR = 0.23, 95% CI = 0.12–0.33, *P* < 0.0001) in blood. HDL-C (*P* = 0.68, I^2^ = 0%) was not heterogeneous among the studies, so fixed-effect model was used to analyzing its OR. Otherwise, random-effect model was used.

### Adverse Events Assessment

**Figure 6:**
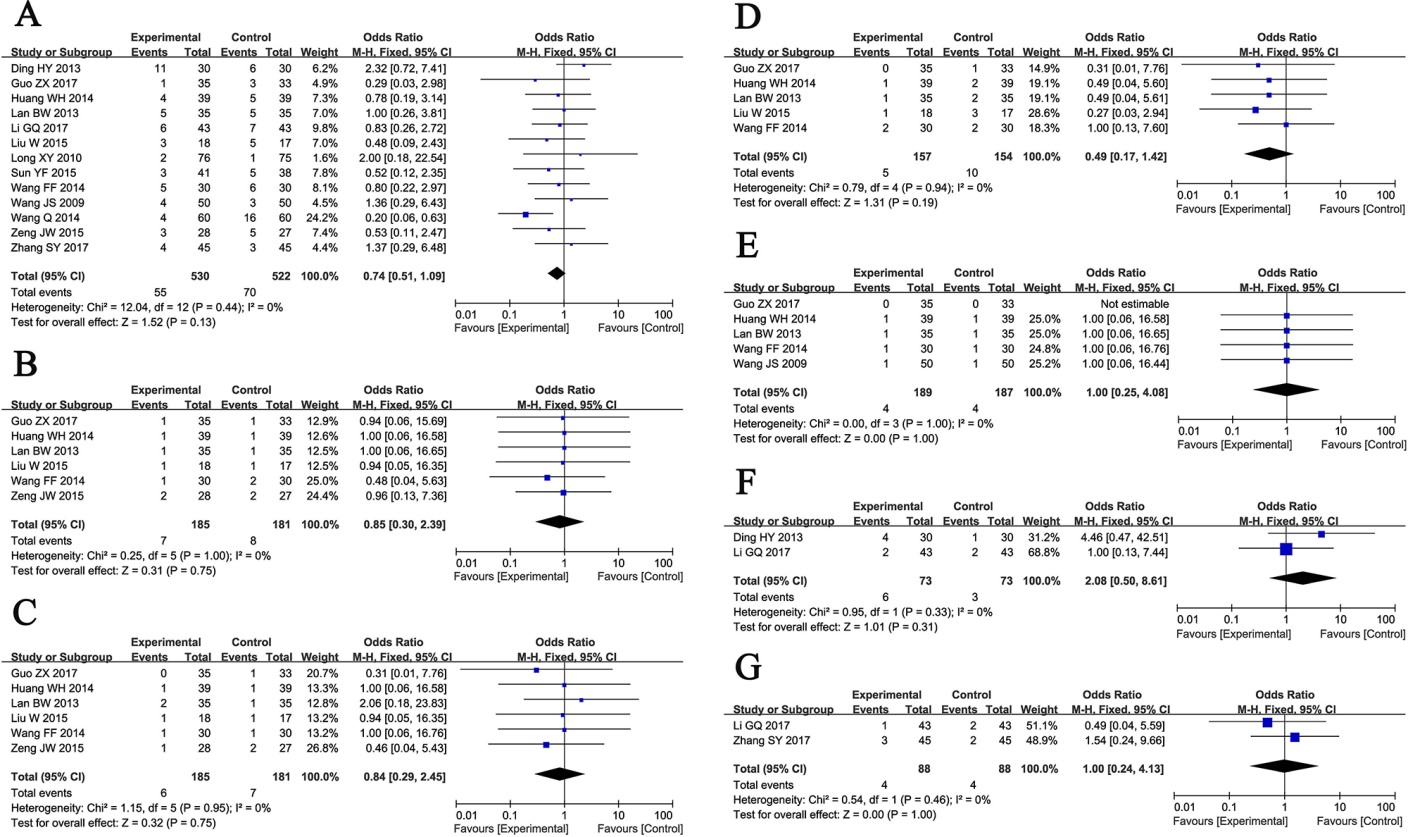
Forest plot of the comparison of adverse effects including total adverse effects **(A)**, fever **(B)**, fullness in head **(C)**, allergy **(D)**, hemorrhage **(E)**, palpitation **(F)**, nausea and vomiting **(G)** between the experimental and control group. Control group, conventional treatments alone group; Experimental group, conventional treatments and GDI combined group; GDI, Ginkgo leaf extract and dipyridamole injection. The fixed-effects meta-analysis model (Mantel-Haenszel method) was used.

Thirteen trials (Wang et al., 2009; Long et al., 2010; Ding, 2013; Lan, 2013; Wang, 2014a; Wang, 2014b; Huang et al., 2014; Liu, 2015; Sun, 2015; Zeng, 2015; Li, 2017; Guo, 2017; Zhang, 2017) involving 1,052 ischemic stroke patients evaluated the safety of GDI mediated therapy. The most common side effects of GDI treatment were fever, fullness in head, allergy, hemorrhage, palpitation, nausea and vomiting which usually subsided after symptomatic treatment. No severe adverse event occurred during GDI treatment, and the occurrence of these adverse reactions in the two groups was similar (**Figure 6**, Total side effects: OR = 0.74, 95% CI = 0.51–1.09, *P* = 0.13; Fever: OR = 0.85, 95% CI = 0.30–2.39, *P* = 0.75; Fullness in head: OR = 0.84, 95% CI = 0.29–2.45, *P* = 0.75; Allergy: OR = 0.49, 95% CI = 0.17–1.42, *P* = 0.19; Hemorrhage: OR = 1.00, 95% CI = 0.25–4.08, *P* = 1.00; Palpitation: OR = 2.08, 95% CI = 0.50–8.61, *P* = 0.31; Nausea and vomiting: OR = 1.00, 95% CI = 0.24–4.13, *P* = 1.00). Fixed-effect models were used to analyze OR rate because of low heterogeneity.

### Publication Bias

**Figure 7:**
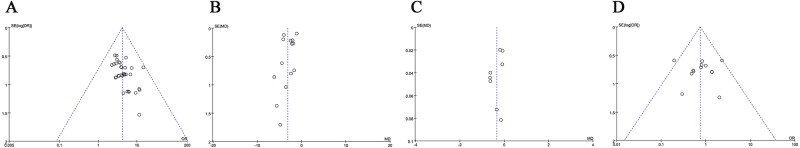
Funnel plot of overall response rate (ORR, **A**), National Institutes of Health Stroke Scale (NIHSS, **B**), plasma viscosity (PV, **C**) and total adverse effects (TAE, **D**).

Publication bias was assessed visually by funnel plots. As shown in **Figure 7**, the funnel plots were symmetrical in NIHSS and total adverse events (TAE), but were asymmetrical in ORR.

We further assessed publication bias by Begg’s and Egger’s regression tests, and ORR was found with bias (Begg = 0.001; Egger = 0.001). No significant publication bias for NIHSS (Begg = 0.584; Egger = 0.638) and total side effects (Begg = 0.855; Egger = 0.986) was observed in these analyses. To determine if the bias affect the pooled risk of ORR, we conducted trim and filled analysis. The adjusted OR indicated same trend with the result of the primary analysis (before: *P* < 0.0001, after: *P* < 0.0001), reflecting the reliability of our primary conclusions.

### Sensitivity Analysis

We also conducted subgroup analysis to explore the source of heterogeneity in ORR, NIHSS and TAE with respect to GDI dosages, sample sizes and types of involved studies. As shown in **Table 2**, our analysis results showed that no significant difference was found between different dosages of GDI, sample sizes and study types in ORR and NIHSS. Moreover, our results showed that GDI may alleviate the TAE caused by routine treatments when its dosage not more than 20 ml/day.

**Table 2:** Subgroup analyses of ORR, NIHSS and TAE between the experimental and control group.

Parameter	Factors at study level	Analysis	Heterogeneity		Odds Ratio	95% CI	*P*-value
		Method	I^2^ (%)	*P*-value	(OR)		
ORR	**Dosage of GDI**						
	20ml/day	Fixed	0	1.00	3.95	3.00–5.21	<0.00001
	>20ml/day	Fixed	0	0.71	4.83	2.97–7.87	<0.00001
	**Study sample size**						
	>80	Fixed	0	1.00	3.41	2.52–4.62	<0.00001
	<80	Fixed	0	0.99	5.53	3.75–8.14	<0.00001
	**Type of control trials**						
	RCTs	Fixed	0	1.00	4.34	3.34–5.64	<0.00001
	Non-RCTs	Fixed	0	0.91	3.30	1.89–5.77	<0.0001
NIHSS	**Dosage of GDI**						
	20ml/day	Fixed	83	<0.00001	−3.01	−3.77 to −2.25	<0.00001
	>20ml/day	Fixed	99	<0.00001	−3.32	−5.07 to −1.57	0.0002
	**Study sample size**						
	>80	Random	94	<0.00001	−3.26	−4.25 to −2.26	<0.00001
	<80	Random	93	<0.00001	−2.94	−3.79 to −2.09	<0.00001
	**Type of control trials**						
	RCTs	Random	98	<0.00001	−3.14	−4.11 to −**2**.18	<0.00001
	Non-RCTs	Random	93	0.0002	−3.16	−5.57 to −0.76 3	0.01
TAE	**Dosage of GDI**						
	20ml/day	Fixed	0	0.64	0.58	0.36–0.91	0.02
	>20ml/day	Fixed	0	0.48	1.40	0.68–2.87	0.36
	**Study sample size**						
	>80	Fixed	41	0.15	0.63	0.35–1.15	0.13
	<80	Fixed	0	0.66	0.83	0.51–1.37	0.46
	**Type of control trials**						
	RCTs	Fixed	19	0.27	0.78	0.51–1.20	0.26
	Non-RCTs	Fixed	0	0.71	0.61	0.26–1.42	0.25

GDI, Ginkgo leaf extract and dipyridamole injection; ORR, overall response rate; NIHSS, National Institutes of Health Stroke Scale; TAE, total adverse events; RCTs, randomized controlled trials.

## Discussion

Traditional Chinese medicine has been used to treat ischemic stroke in China during the past two thousand years. A survey in China showed that about 70% of doctors surveyed indicated that Chinese herb were effective complementary therapies for ischemic stroke (Zeng et al., 2005). GDI, a kind of Chinese medicine preparation, has been clinically applied as an effective adjuvant agent for reducing brain injuries, and enhancing functional recovery (Nash and Shah, 2015; Wang et al., 2015; Li et al., 2017). Even though there was statistical analysis of published literatures, a comprehensive and systematic evaluation of GDI for the treatment of ischemia stroke is still rare. In this analysis, we conducted a wide range of online search according strict inclusion and exclusion criteria, by which to provide an internationally accessible systematic review of the clinical efficacy and safety of GDI for the ischemia stroke.

The meta-analysis was carried out in thirty-five articles (Tang et al., 2009; Wang et al., 2009; Tian et al., 2010; Long et al., 2010; Zhou et al., 2010; Chen et al., 2011; Wang, 2012; Yang, 2012; Tang, 2013; Lan, 2013; Lin and Lin, 2013; Huang et al., 2014; Fang, 2014; Jiang, 2014; Wang, 2014a; Wang, 2014b; Yang, 2014; Liu, 2015; Zeng, 2015; Sun, 2015; Chen, 2016; Li, 2016; Zhou et al., 2016; Cui, 2016; Fu et al., 2016; Wei, 2017; Guo, 2017; Ai, 2017; Zhang, 2017; Dai, 2017; Li, 2017; Wang, 2018; Yi et al., 2018; Zhang, 2018; Zheng et al., 2018) to evaluate the ORR. Compared with conventional treatments alone, GDI combined with conventional treatments was associated with obviously higher ORR. Moreover, the combination therapy also significantly improved the neurological status and activity of daily living of ischemic stroke patients. CRP has important value in the prediction, prevention and prognosis of ischemic stroke (Matsuo et al., 2016; Yu et al., 2019). Our analysis results showed that the CRP level of patients was obviously decreased after conventional treatments and GDI combined therapy. Moreover, hemorheology and blood lipids indexes of patients were also significantly ameliorated. All these results indicated that GDI can protect ischemic stroke from injury, which may be related with its action on regulating the blood viscosity and level of blood lipid.

Safety is the top priority of a therapeutic strategy, and also a key factor for their clinical application and further development. This analysis confirmed the safety of GDI in ischemic stroke treatment. The most common side effects during GDI therapy were fever, fullness in head, allergy, hemorrhage, palpitation, nausea, and vomiting, and all of them did not differ significantly between the two groups. Therefore, GDI is a safe auxiliary medicine for ischemic stroke.

The analysis on therapeutic effects may be influenced by several factors. We used three clinical variables (GDI dosages, sample sizes, and research types) to interact with three outcome indicators (ORR, NIHSS, and TAE) and found that the TAE might be associated with GDI dosages. However, recent studies on the impact of these factors on the curative effect of GDI adjuvant therapy remain insufficient and further investigations such as biobliographic references that support this statement still should be performed.

There are some limitations in our analysis. As an important Chinese herb preparation, GDI was mainly applied in China, which may bring the unavoidable regional bias and subsequently influence the clinical application of GDI worldwide. In other countries such as America, India and Iran, *G. biloba* extract has also been used for acute ischemic stroke treatment and the prevention of cognitive decline (Garg et al., 1995; Dodge et al., 2008; Oskouei et al., 2013), but these studies were eventually excluded because they did not meet the inclusion criteria. We will keep following the updated researches on GDI mediated therapy for ischemic stroke in the world, and perform further systematical research on it. Moreover, several results showed significantly heterogeneity among the included trials, which may be due to the different ages of ischemic stroke patients and routine drug types. Therefore, the findings from our study should be dealt with some caution. Finally, a possible interaction between other drugs and ginkgo should be considered. However, our data were extracted from published papers and they did not provide sufficient information on this aspect. Therefore, based on currently available literatures, there are insufficient data to perform a statistical analysis to evaluate the correlation. We will keep paying close attention to this concern in our later studies

## Conclusion

In summary, this meta-analysis indicated that GDI combined with conventional treatments was effective in treating ischemic stroke. Clinical application of GDI not only obviously enhanced the ORR of conventional treatments, but also effectively improved the blood viscosity and blood lipid level of ischemic stroke patients. However, because the low quality of some included trials increases risks and bias, the clinical efficacy and safety of GDI-mediate therapy for ischemic stroke still needs methodologically rigorous trials to verify.

## Data Availability Statement

All datasets generated for this study are included in the article/**Supplementary Material**.

## Author Contributions

SL and ZM put forward this topic and designed this study. PX and ZM performed article screening, data collection and extraction, and manuscript writing. PX, ZM, and SL conducted the data analysis. SL polished the written English.

## Conflict of Interest

The authors declare that the research was conducted in the absence of any commercial or financial relationships that could be construed as a potential conflict of interest.

## Supplementary Material

Supplementary Figure 1 Sensitivity Analysis for National Institutes of Health Stroke Scale (NIHSS).

Supplementary Figure 2 Forest plot of the comparison of plasma C reactive protein (CRP) between the experimental and control group. Control group, conventional treatments alone group; Experimental group, conventional treatments and GDI combined group. GDI, Ginkgo leaf extract and dipyridamole injection. The random effects meta-analysis model (Inverse Variance method) was used.

Supplementary Figure 3 Forest plot of the comparison of the hemorrheology indexes including WBV **(A)**, PV **(B)**, WBHSV **(C)**, WBLSV **(D)** and FIB **(E)** between the experimental and control group. Control group, conventional treatments alone group; Experimental group, conventional treatments and GDI combined group. WBV, whole blood viscosity; PV, plasma viscosity; WBHSV, whole blood high-shear viscosity; WBLSV, whole blood low-shear viscosity; FIB, content of fibrinogen; GDI, Ginkgo leaf extract and dipyridamole injection.

Supplementary Figure 4 Forest plot of the comparison of the blood lipid indexes including TC **(A)**, TG **(B)**, HDL-C **(C)** and LDL-C **(D)** between the experimental and control group. Control group, conventional treatments alone group; Experimental group, conventional treatments and GDI combined group. TC, plasma total cholesterol; TG, triglycerides; HDL-C, high density lipoprotein-cholesterol; LDL-C, low density lipoprotein-cholesterol; GDI, Ginkgo leaf extract and dipyridamole injection.
