# Supplementary figures and images for: Efficacy and Safety of Ginkgo Leaf Extract and Dipyridamole Injection for Ischemic Stroke: A Systematic Review and Meta Analysis
Source: Front Pharmacol. 2019 Dec 4;10:1403. doi: 10.3389/fphar.2019.01403 (PMC6904941; doi:10.3389/fphar.2019.01403)

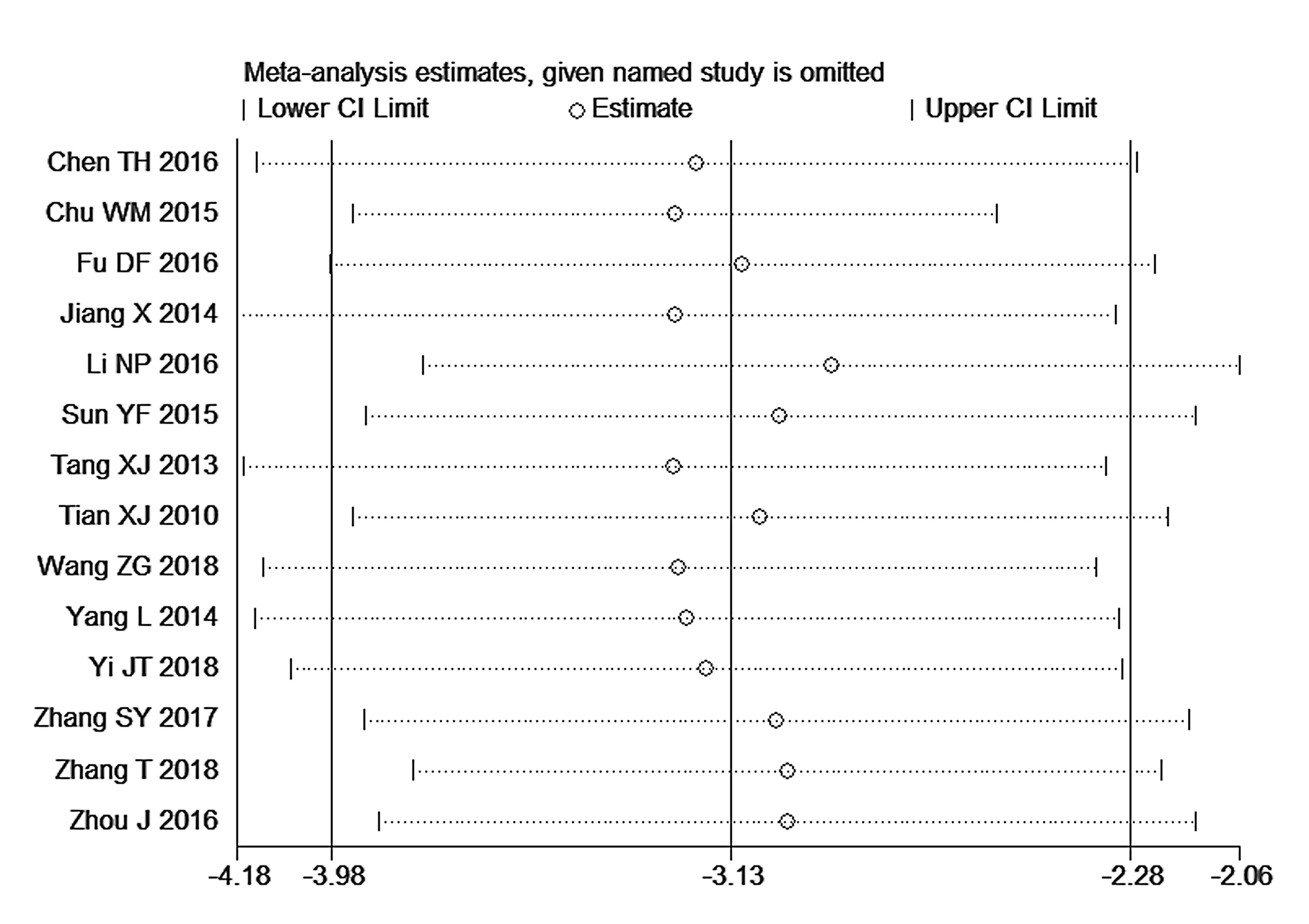

Supplement: Supplementary Figure 1 — Sensitivity Analysis for National Institutes of Health Stroke Scale (NIHSS). [file Image_1.jpeg]

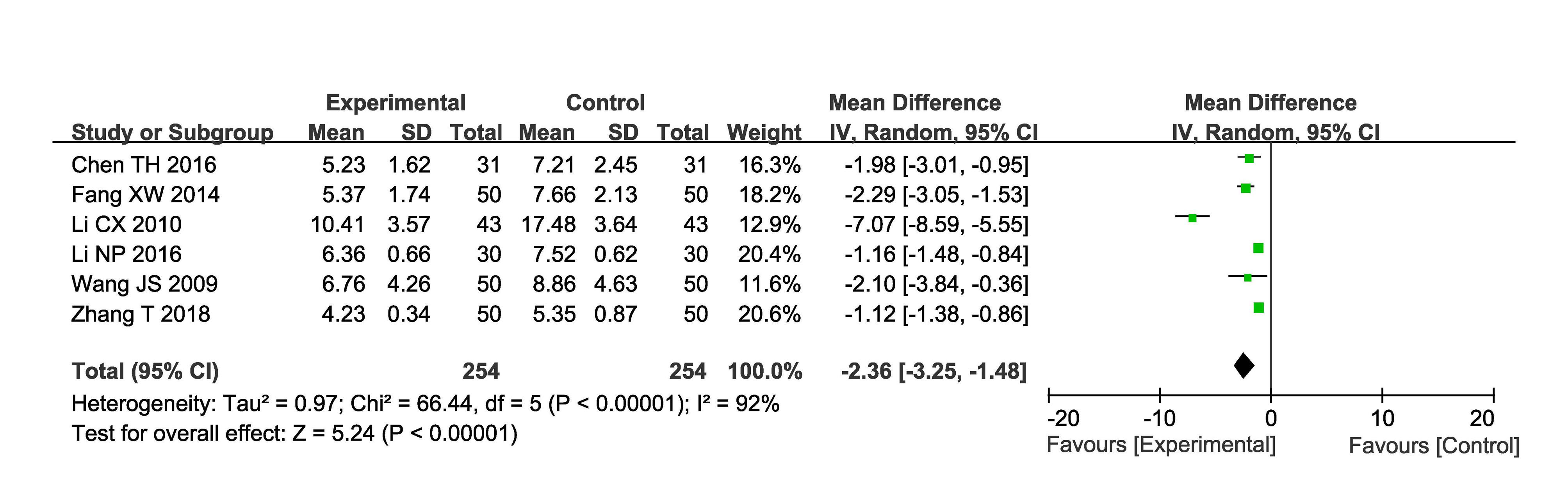

Supplement: Supplementary Figure 2 — Forest plot of the comparison of plasma C reactive protein (CRP) between the experimental and control group. Control group, conventional treatments alone group; Experimental group, conventional treatments and GDI combined group. GDI, Ginkgo leaf extract and dipyridamole injection. The random effects meta-analysis model (Inverse Variance method) was used. [file Image_2.jpeg]

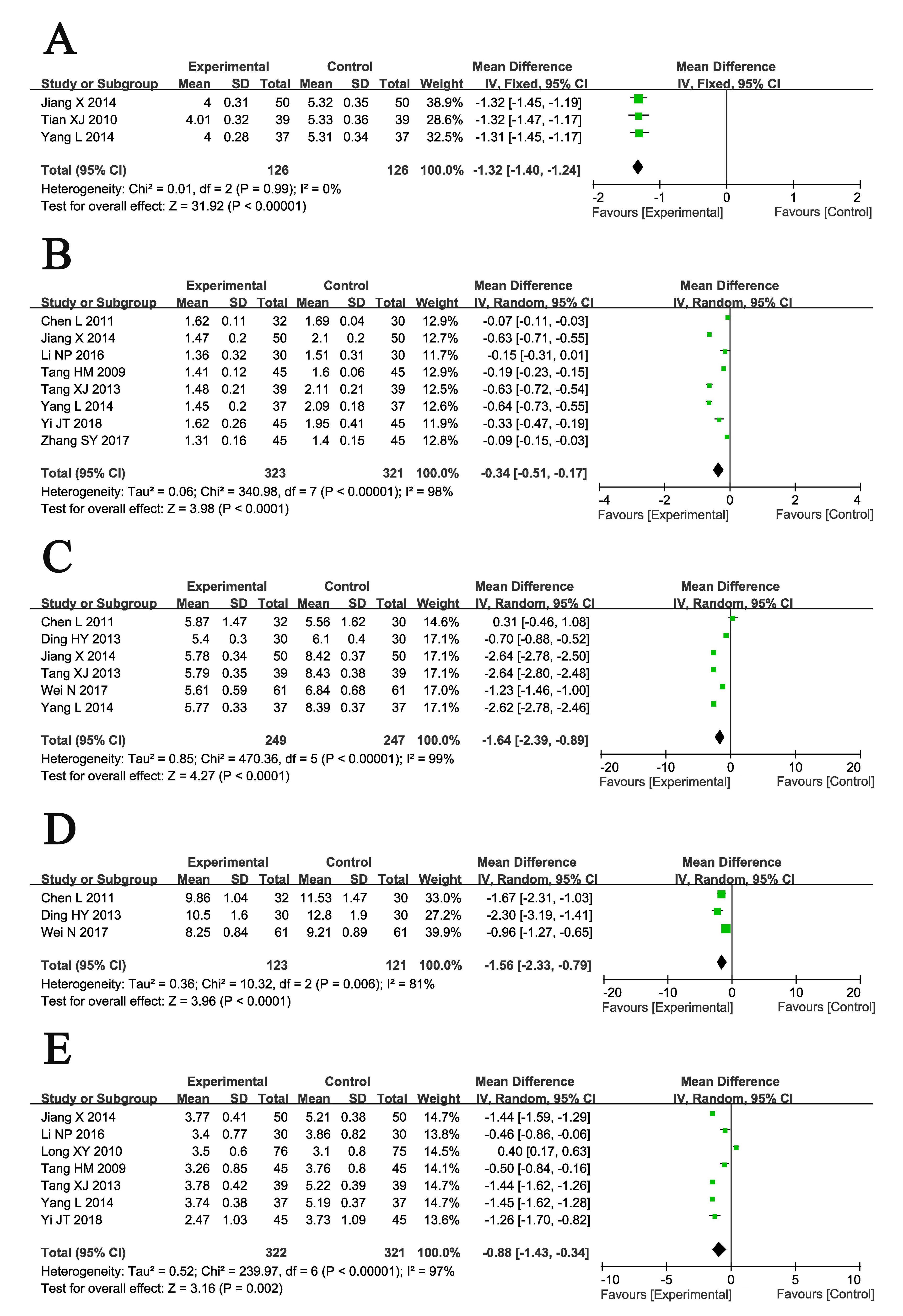

Supplement: Supplementary Figure 3 — Forest plot of the comparison of the hemorrheology indexes including WBV (A), PV (B), WBHSV (C), WBLSV (D) and FIB (E) between the experimental and control group. Control group, conventional treatments alone group; Experimental group, conventional treatments and GDI combined group. WBV, whole blood viscosity; PV, plasma viscosity; WBHSV, whole blood high-shear viscosity; WBLSV, whole blood low-shear viscosity; FIB, content of fibrinogen; GDI, Ginkgo leaf extract and dipyridamole injection. [file Image_3.jpeg]

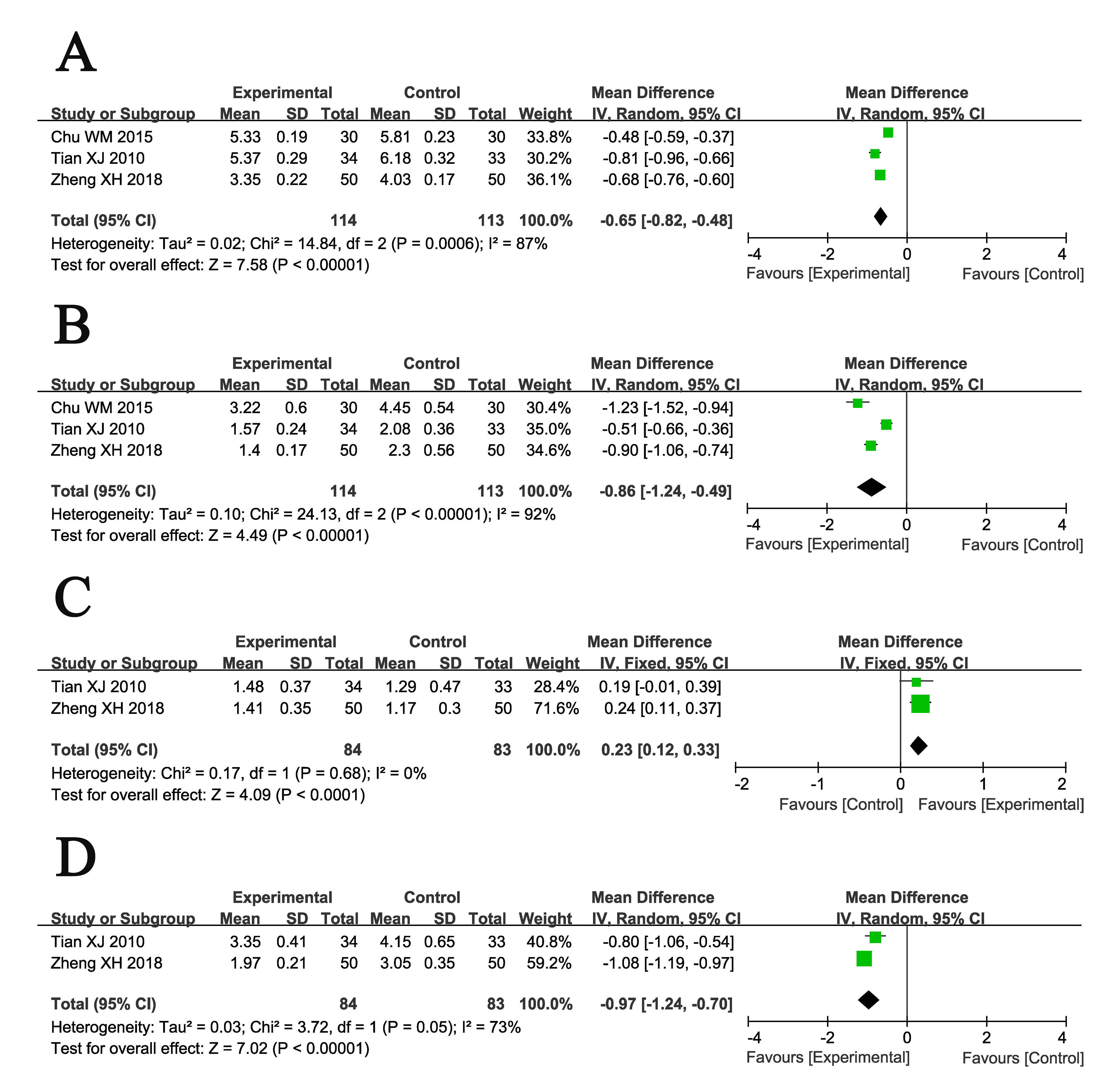

Supplement: Supplementary Figure 4 — Forest plot of the comparison of the blood lipid indexes including TC (A), TG (B), HDL-C (C) and LDL-C (D) between the experimental and control group. Control group, conventional treatments alone group; Experimental group, conventional treatments and GDI combined group. TC, plasma total cholesterol; TG, triglycerides; HDL-C, high density lipoprotein-cholesterol; LDL-C, low density lipoprotein-cholesterol; GDI, Ginkgo leaf extract and dipyridamole injection. [file Image_4.jpeg]
