# Supplementary material for: Efficacy and Safety of Ginkgo Leaf Extract and Dipyridamole Injection for Ischemic Stroke: A Systematic Review and Meta Analysis
Source: Front Pharmacol. 2019 Dec 4;10:1403. doi: 10.3389/fphar.2019.01403 (PMC6904941; doi:10.3389/fphar.2019.01403)
Supplement: Supplementary file 5 [file Table_1.doc]

**Supplementary Table 1.** Searching strategy for electronic databases

| **Data base** | **Search Strategy** |
| --- | --- |
| **English database:** PubMed, Cochrane Library, Medline, Embase and Web of Science. | **#1.** cerebrovascular disorders or stroke or ischemic stroke or cerebral infarction or brain infarction or Brain Ischemia or cerebral ischemia or intracranial arterial diseases or Carotid Artery Diseases or Intracranial Arterial Diseases or Intracranial Embolism [Title/Abstract].  **#2.** Brain Ischemia [MeSH].  **#3.** #1 or #2.  **#4.** yinxing or yinxingtiquwu or ginkgo biloba or ginkgo biloba extract or ginkgo leaf extract or yinxingdamo or ginkgo dipyidamolum or ginkgo leaf extract and dipyridamole [Title/Abstract].  **#5.** injection.  **#6.** #3 and #4 and #5  **#7.** limit #6 to human  **#8.** limit #7 to (controlled clinical trial)  **#9.** limit #8 to yr="2009-Current" |
| **Chinese database:** Wanfang database, Chinese Scientific Journal Database (VIP), China National Knowledge Infrastructure (CNKI) and Chinese Biological Medicine Database (CBM). | **#1.** cuzhong (stroke) or naocuzhong (stroke) or zhongfeng (stroke) or naozhongfeng (stroke ) or quexuexingnaoxueguanbing (ischemic cerebrovascular disease) or quexuexingcuzhong  (ischemic stroke) or quexuexingnaocuzhong (ischemic stroke) or quexuexingzhongfeng (ischemic stroke ) or quexuexingnaozhongfeng (ischemic stroke ) or naogengsi (brain infarction) or naogengsai (brain infarction) [Title/Keywords].  **#2.** yinxing (ginkgo biloba) or yinxingtiquwu (ginkgo biloba extract) or yinxingyetiquwu (ginkgo leaf extract) or yinxingdamo (ginkgo dipyidamolum)[Title/Keywords].  **#3.** zhusheye or zhusheji [Title/Keywords].  **#4.** #1 and #2 and #3.  **#5.** limit #4 to human  **#6.** limit #5 to (controlled clinical trial)  **#7.** limit #6 to yr="2009-Current" |
