# Supplementary material for: Efficacy and Safety of Ginkgo Leaf Extract and Dipyridamole Injection for Ischemic Stroke: A Systematic Review and Meta Analysis
Source: Front Pharmacol. 2019 Dec 4;10:1403. doi: 10.3389/fphar.2019.01403 (PMC6904941; doi:10.3389/fphar.2019.01403)
Supplement: Supplementary file 6 [file Table_2.doc]

**Supplementary Table 2.** Manufacturer information of Ginkgo leaf extract and dipyridamole injection (GDI)

| Included studies | Manufacturer | Manufacturing Approve Number |
| --- | --- | --- |
| Ai DJ 2017 | Not given |  |
| Chen L 2011 | Meihekou Sihuan Pharmaceutical Co., Ltd. | H22026140 |
| Chen TH 2016 | Shanghai New Pioneer Pharmaceutical Co., Ltd. | H20045315 |
| Chu WM 2015 | Not given |  |
| Cui XF 2016 | Not given |  |
| Dai CM 2017 | Hubei Minkang Pharmaceutical Co., Ltd. | H42022869 |
| Ding HY 2013 | Shanghai New Pioneer Pharmaceutical Co., Ltd. | H20045315 |
| Fang XW 2014 | Guizhou Yibai Pharmaceutical Co., Ltd. | H52020031 and H52020032 |
| Fu DF 2016 | Shanxi Pude Pharmaceutical Co., Ltd. | H14023515 |
| Guo ZX 2017 | Guizhou Yibai Pharmaceutical Co., Ltd. | H52020031 and H52020032 |
| Huang SP 2010 | Shanghai New Pioneer Pharmaceutical Co., Ltd. | H20045315 |
| Huang WH 2014 | Not given |  |
| Jiang X 2014 | Hubei Minkang Pharmaceutical Co., Ltd. | H42022869 |
| Lan BW 2013 | Shanxi Pude Pharmaceutical Co., Ltd. | H14023515 |
| Li CX 2010 | Not given |  |
| Li GQ 2017 | Guizhou Yibai Pharmaceutical Co., Ltd. | H52020031 and H52020032 |
| Li NP 2016 | Not given |  |
| Lin CD 2013 | Meihekou Sihuan Pharmaceutical Co., Ltd. | H22026140 |
| Liu W 2015 | Not given |  |
| Long XY 2010 | Not given |  |
| Sun YF 2015 | Guizhou Yibai Pharmaceutical Co., Ltd. | H52020031 and H52020032 |
| Tang HM 2009 | Shanxi Pude Pharmaceutical Co., Ltd. | H14023515 |
| Tang XJ 2013 | Not given |  |
| Tian XJ 2010 | Shanxi Pude Pharmaceutical Co., Ltd. | H14023515 |
| Wang FF 2014 | Not given |  |
| Wang JS 2009 | Hubei Minkang Pharmaceutical Co., Ltd. | H42022869 |
| Wang Q 2014 | Not given |  |
| Wang YH 2012 | Not given |  |
| Wang ZG 2018 | Guizhou Yibai Pharmaceutical Co., Ltd. | H52020031 and H52020032 |
| Wei N 2017 | Shanxi Pude Pharmaceutical Co., Ltd. | H14023515 |
| Yang J 2012 | Not given |  |
| Yang L 2014 | Not given |  |
| Yi JT 2018 | Shanxi Pude Pharmaceutical Co., Ltd. | H14023515 |
| Zeng JW 2015 | Guizhou Yibai Pharmaceutical Co., Ltd. | H52020031 and H52020032 |
| Zhang SY 2017 | Not given |  |
| Zhang T 2018 | Guizhou Yibai Pharmaceutical Co., Ltd. | H52020031 and H52020032 |
| Zheng XH 2018 | Not given |  |
| Zhou J 2016 | Guizhou Yibai Pharmaceutical Co., Ltd. | H52020031 and H52020032 |
| Zhou M 2010 | Not given |  |
