# Supplementary material for: Efficacy and Safety of Ginkgo Leaf Extract and Dipyridamole Injection for Ischemic Stroke: A Systematic Review and Meta Analysis
Source: Front Pharmacol. 2019 Dec 4;10:1403. doi: 10.3389/fphar.2019.01403 (PMC6904941; doi:10.3389/fphar.2019.01403)
Supplement: Supplementary file 7 [file Table_3.doc]

**Supplementary Table 3.** The results of sensitivity analysis of clinical trials.

| **Study omitted** | **Estimate** | **[95% Conf. interval]** | |
| --- | --- | --- | --- |
|  |  | Lower CI limit | Upper CI limit |
| Chen TH 2016 | -3.21 | -4.14 | -2.27 |
| Chu WM 2015 | -3.25 | -3.93 | -2.57 |
| Fu DF 2016 | -3.11 | -3.98 | -2.23 |
| Jiang X 2014 | -3.25 | -4.18 | -2.32 |
| Li NP 2016 | -2.92 | -3.78 | -2.06 |
| Sun YF 2015 | -3.03 | -3.91 | -2.15 |
| Tang XJ 2013 | -3.25 | -4.17 | -2.34 |
| Tian XJ 2010 | -3.07 | -3.93 | -2.21 |
| Wang ZG 2018 | -3.24 | -4.13 | -2.36 |
| Yang L 2014 | -3.23 | -4.14 | -2.31 |
| Yi JT 2018 | -3.19 | -4.07 | -2.30 |
| Zhang SY 2017 | -3.04 | -3.91 | -2.16 |
| Zhang T 2018 | -3.01 | -3.81 | -2.22 |
| Zhou J 2016 | -3.01 | -3.88 | -2.15 |
| Combined | -3.13 | -3.98 | -2.28 |
